# Noninvasive assessment of airflows by electrical impedance tomography in intubated hypoxemic patients: an exploratory study

**DOI:** 10.1186/s13613-019-0560-5

**Published:** 2019-07-22

**Authors:** Tommaso Mauri, Elena Spinelli, Francesca Dalla Corte, Eleonora Scotti, Cecilia Turrini, Marta Lazzeri, Laura Alban, Marco Albanese, Donatella Tortolani, Yu-Mei Wang, Savino Spadaro, Jian-Xin Zhou, Antonio Pesenti, Giacomo Grasselli

**Affiliations:** 1 Department of Anesthesia, Critical Care and Emergency, Fondazione IRCCS Ca’ Granda Ospedale Maggiore Policlinico, Via F. Sforza 35, 20122 Milan, Italy; 2 Department of Pathophysiology and Transplantation, University of Milan, Milan, Italy; 3 Department of Morphology, Surgery and Experimental Medicine, Azienda Ospedaliera-Universitaria Arcispedale Sant’Anna, University of Ferrara, Ferrara, Italy; 4 Department of Critical Care Medicine, Beijing Tiantan Hospital, Capital Medical University, Beijing, China

**Keywords:** Electrical impedance, Spirometry, Respiratory airflow, Mechanical ventilation, Acute respiratory distress syndrome, Respiratory failure

## Abstract

**Background:**

Noninvasive monitoring of maximal inspiratory and expiratory flows (MIF and MEF, respectively) by electrical impedance tomography (EIT) might enable early recognition of changes in the mechanical properties of the respiratory system due to new conditions or in response to treatments. We aimed to validate EIT-based measures of MIF and MEF against spirometry in intubated hypoxemic patients during controlled ventilation and spontaneous breathing. Moreover, regional distribution of maximal airflows might interact with lung pathology and increase the risk of additional ventilation injury. Thus, we also aimed to describe the effects of mechanical ventilation settings on regional MIF and MEF.

**Methods:**

We performed a new analysis of data from two prospective, randomized, crossover studies. We included intubated patients admitted to the intensive care unit with acute hypoxemic respiratory failure (AHRF) and acute respiratory distress syndrome (ARDS) undergoing pressure support ventilation (PSV, *n* = 10) and volume-controlled ventilation (VCV, *n* = 20). We measured MIF and MEF by spirometry and EIT during six different combinations of ventilation settings: higher vs. lower support during PSV and higher vs. lower positive end-expiratory pressure (PEEP) during both PSV and VCV. Regional airflows were assessed by EIT in dependent and non-dependent lung regions, too.

**Results:**

MIF and MEF measured by EIT were tightly correlated with those measured by spirometry during all conditions (range of *R*^2^ 0.629–0.776 and *R*^2^ 0.606–0.772, respectively, *p* < 0.05 for all), with clinically acceptable limits of agreement. Higher PEEP significantly improved homogeneity in the regional distribution of MIF and MEF during volume-controlled ventilation, by increasing airflows in the dependent lung regions and lowering them in the non-dependent ones.

**Conclusions:**

EIT provides accurate noninvasive monitoring of MIF and MEF. The present study also generates the hypothesis that EIT could guide PSV and PEEP settings aimed to increase homogeneity of distending and deflating regional airflows.

**Electronic supplementary material:**

The online version of this article (10.1186/s13613-019-0560-5) contains supplementary material, which is available to authorized users.

## Introduction

Electrical impedance tomography (EIT) is a noninvasive, bedside, radiation-free, dynamic lung imaging technique. EIT provides intrathoracic maps of lung impedance changes referenced to a baseline (i.e., the end-expiratory lung volume from previous breath) every 20–50 ms [1]. Intrathoracic impedance changes measured by EIT are linearly correlated with global and regional tidal volume, and the correlation is maintained at increasing positive end-expiratory pressure (PEEP) levels [2]. Thus, EIT yields noninvasive bedside continuous measure of regional lung volume changes during inspiration and expiration.

Inspiratory and expiratory airflows correspond to the velocity of lung volume changes over time. In intubated patients, they are traditionally measured through a spirometer applied to the ventilator circuit before the endotracheal tube or within the ventilator. Global maximal inspiratory and expiratory flows (MIF and MEF, respectively) measured by standard spirometry depend on the mechanical properties of the respiratory system (namely, lung compliance and airway resistance) [3]. Therefore, monitoring of MIF and MEF could be useful to guide ventilation settings (e.g., by selecting the positive pressure level associated with improved mechanics) and/or to evaluate the efficacy of pharmacologic treatments (e.g., increased MIF and/or MEF after bronchodilator drugs) [4]. However, spirometry only yields global measures of MIF and MEF, while heterogeneous distribution of altered lung mechanics is a hallmark of acute hypoxemic respiratory failure (AHRF) and acute respiratory distress syndrome (ARDS) [5]. Alveolar damage leads to collapse of lung units tightly bordering normal-, partial- and over-inflated units, potentially yielding imbalances in regional MIF and MEF values. Such imbalances can increase the risk of ventilator-induced lung injury (VILI) through multiple mechanisms [6], while settings obtaining more homogenous regional flows might reduce it. External classic spirometry sometimes leads to altered respiratory patterns and inaccurate measures, too [7]. Thus, a noninvasive bedside dynamic method to measure global and regional MIF and MEF values would be a valuable addition in understanding AHRF and ARDS patients’ pathophysiology and to guide personalized treatments.

In the present study, following preliminary data obtained in animal model [8], we aimed to validate in intubated AHRF and ARDS patients undergoing controlled ventilation and spontaneous breathing EIT-based measures of global MIF and MEF against standard spirometry. Moreover, we explored the effects of higher vs. lower PEEP and pressure support levels on regional flows; our hypothesis is that higher PEEP and lower pressure support could yield more homogenous distribution of regional MIF and MEF.

## Materials and methods

### Study population

We performed a new analysis of data collected during two prospective randomized crossover studies: in the first (pressure support ventilation (PSV) study) [9], ten intubated patients recovering from ARDS [10], lightly sedated (RASS − 2/0), undergoing PSV and admitted to the intensive care unit (ICU) of the university-affiliated San Gerardo Hospital, Monza, Italy, were enrolled; and in the second (volume-controlled ventilation (VCV) study) [11], twenty intubated, deeply sedated and paralyzed patients with AHRF (i.e., PaO_2_/FiO_2_ ≤ 300, PEEP ≥ 5 cmH_2_O, acute onset, no cardiac failure) or ARDS admitted to the same ICU were enrolled. The ethical committee of San Gerardo Hospital, Monza, Italy, approved the studies, and informed consent was obtained following local regulations. Additional details on the inclusion and exclusion criteria for the two studies are provided in an online data supplement (Additional file 1).

### Demographic data collection

We collected sex, age, Simplified Acute Physiology Score II values, etiology, diagnosis and severity of ARDS, days on mechanical ventilation before study enrollment for each patient. In-hospital mortality was recorded, too.

### EIT and ventilation monitoring

In each patient, EIT-dedicated belt, containing 16 equally spaced electrodes, was placed around the thorax at the fifth or sixth intercostal space and connected to a commercial EIT monitor (PulmoVista 500, Dräger Medical GmbH, Lübeck, Germany). During all study phases, EIT data were generated by application of small alternate electrical currents rotating around patient’s thorax, continuously recorded at 20 Hz and stored for offline analysis, as previously described [12]. Synchronized to EIT tracings, airway pressure and airflows from the mechanical ventilator were continuously recorded.

### Interventions

More details on the two protocols can be found in the online data supplement (Additional file 1).

Briefly, in the PSV study, patients underwent the following crossover randomized steps, each lasting 20 min:

1. Low support at clinical PEEP (PSV_low_) vs. higher support at clinical PEEP (PSV_high_);
2. Clinical support at low PEEP (PSV-PEEP_low_) vs. clinical support at higher PEEP (PSV-PEEP_high_).

In the VCV study, instead, the following phases were performed in crossover randomized order, each lasting 20 min:

1. Protective VCV at low PEEP (VCV-PEEP_low_) vs. protective VCV at clinical PEEP + 5 cmH_2_O (VCV-PEEP_high_).

### EIT and ventilation data

**Fig. 1:**
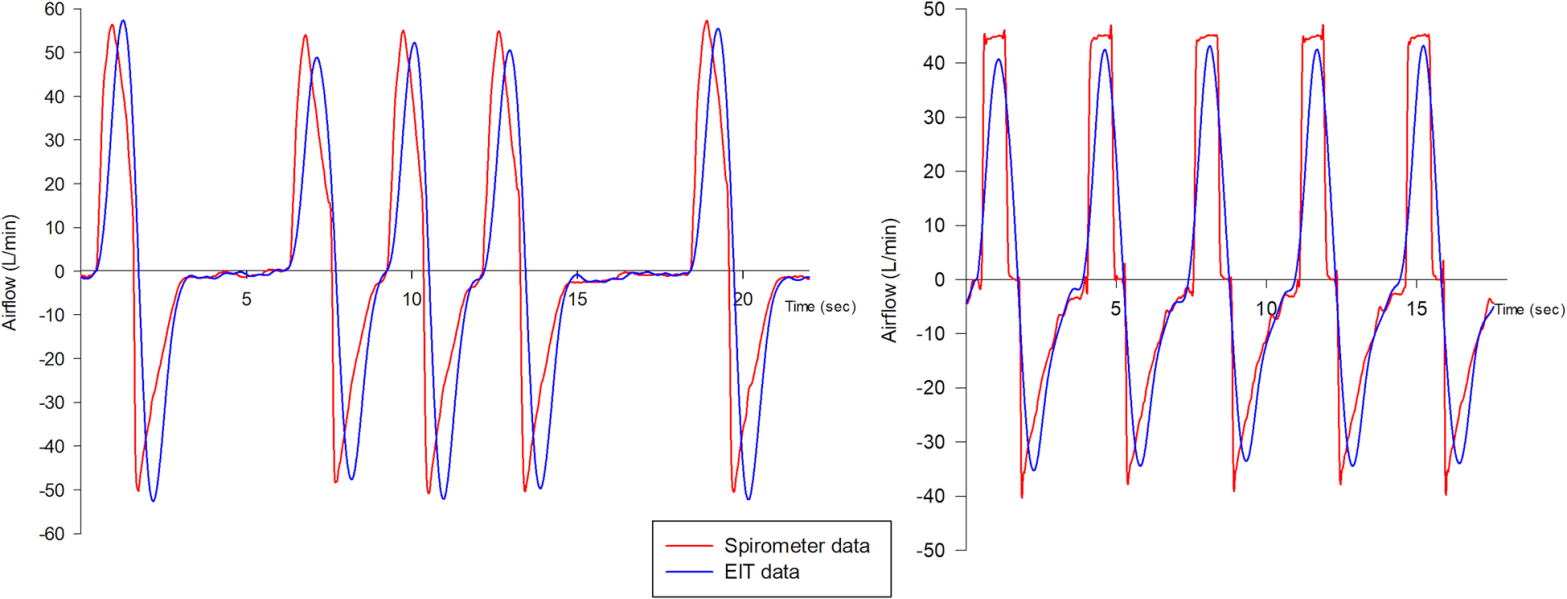
Airflow waveforms measured by spirometer (red line) and electrical impedance tomography (EIT) (blue line) in two representative patients during pressure support ventilation (left) and volume-controlled ventilation (right). Note the correspondence between peak values by the two methods, with only modest underestimation by EIT

From offline analysis of the EIT tracings obtained during the last minutes of each phase (analysis of ten breaths), we measured global and regional (same-size dependent and non-dependent lung regions) noninvasive airflows waveform, as previously described [8]. Briefly, instantaneous global and regional inspiratory and expiratory airflows were measured as variations of global and regional impedance measured every 50 ms, multiplied by the tidal volume/tidal impedance ratio from the same study phase and divided by 50 ms. EIT airflow data were then transformed from mL/msec to L/min (Fig. 1), and the maximum EIT-derived global and regional MIF and MEF (MIF_glob_, MIF_non-dep_ and MIF_dep_; MEF_glob_, MEF_non-dep_ and MEF_dep_, respectively) were identified and the value averaged over 5–10 consecutive respiratory cycles.

Regional index of flow homogeneity for both MIF and MEF was then calculated as the ratio between regional flow in the non-dependent and dependent regions (i.e. MIF_non-dep/dep_ and MEF_non-dep/dep_). Values nearer to 1 unit indicate more homogenous distribution of regional airflows.

At the same time points of EIT data analysis, waveforms from the ventilator spirometer were analyzed to measure global MIF and MEF (MIF_spiro_ and MEF_spiro_, respectively) and the value averaged over ten consecutive respiratory cycles. In one patient from the PSV study, technical issues with stored recordings prevented accurate measure of MIF_spiro_ and MEF_spiro_.

Of note, in the present study, the terms MIF and MEF indicated the maximum inspiratory and expiratory flows measured under standardized settings in intubated patients undergoing PSV and VCV at different PEEP levels. In classic physiology, instead, MIF and MEF are measured during volitional maximum efforts. Indeed, during VCV, we did not perform additional volitional maneuvers for measuring MIF and MEF but rather compared airflows measured by EIT under standardized settings. Finally, expiratory flow is usually reported as negative value, but for the sake of clarity, in tables and results we expressed it as absolute values.

Finally, at the same time points, Global Inhomogeneity Index (GI) of the distribution of regional tidal volume was calculated as previously described [13].

### Statistical analysis

Normal distribution was tested by the Shapiro–Wilk test. Data are presented as the mean ± standard deviation (SD) or median and interquartile range [IQR] for continuous variables, as appropriate. Absolute or relative frequencies (%) are used for categorical variables. Comparisons between two groups of normally distributed variables were performed by repeated-measure *t* test, while nonnormally distributed variables were compared by Wilcoxon signed rank test. Differences in categorized variables were assessed using the Chi-square test or the Fisher’s exact test, as appropriate. Association between MIF and MEF obtained by EIT and spirometry was assessed by linear regression. Agreement between measures of MIF and MEF assessed by EIT and spirometry was assessed by Bland–Altman plots. A level of *p* < 0.05 (two-tailed) was considered as statistically significant. Statistical analyses were performed by using Sigma-Plot 12.0 (Systat Software Inc., San Jose, CA, USA).

## Results

### Patients’ characteristics and mechanical ventilation settings

Patients’ characteristics are listed in Table 1: patients were relatively young and quite severe, with short delay between intubation and enrollment of this study (early AHRF and ARDS). Patients with ARDS on the day of the study were 5 in the PSV study vs. 8 in the VCV study (50% vs. 40%, *p* = 0.602) but PaO_2_/FiO_2_ and number of quadrants involved were more severe in the VCV study group (Table 1).

**Table 1:** Main characteristics of the study population

Patients characteristics	All patients (*n* = 30)	PSV study (*n* = 10)	VCV study (*n* = 20)	*p* value PSV study vs. VCV study
Age, years	61 ± 12	60 ± 10	62 ± 13	0.729
Male sex, n (%)	17 (57)	5 (50)	12 (60)	0.602
SAPS II score at admission	50 ± 17	54 ± 19	48 ± 15	0.361
Etiology of AHRF, n (%)				0.168
Pneumonia	16 (53)	5 (50)	11 (55)	
Septic shock	6 (20)	1 (10)	5 (25)	
Trauma	2 (7)	2 (20)	0	
Postoperative AHRF	3 (10)	2 (20)	1 (5)	
Other	3 (10)	0 (0)	3 (15)	
ARDS on the day of the study, n (%)	13 (43)	5 (50)	8 (40)	0.602
Number of lung quadrants involved, n	2 [1–3]	2 [1, 2]	3 [2–4]	0.035
PaO_2_/FiO_2_	213 ± 56	241 ± 46	199 ± 57	0.053
Days on MV before study, n	1 [1–2]	2 [2–4]	1 [1–2]	0.055
In-hospital mortality, n (%)	9 (30)	2 (20)	7 (35)	0.398

PSV, pressure support ventilation; VCV, volume-controlled ventilation; SAPS, simplified acute physiology score; AHRF, acute hypoxemic respiratory failure; ARDS, adult respiratory distress syndrome; PaO_2_/FiO_2_, oxygen partial arterial tension/inspired oxygen fraction; MV, mechanical ventilation

In the PSV study, lower support (PSV_low_) was 3 ± 3 cmH_2_O while PSV_high_ was 12 ± 3 cmH_2_O (*p *< 0.0001), both applied at clinical PEEP level of 7 ± 2 cmH_2_O. Tidal volume increased between the two phases (6.8 [5.2–8.2] mL/kg vs 8.2 [6.5–12.3] mL/kg, *p* = 0.005).

In the same study, lower PEEP (PSV-PEEP_low_) level was 7 ± 2 cmH_2_O, while PSV-PEEP_high_ was 12 ± 2 cmH_2_O (*p *< 0.0001), both implemented with clinical support of 8 ± 5 cmH_2_O. Tidal volume remained stable over these two phases (8.2 [6.7–10.2] mL/kg vs. 7.8 [6.4–10.7] mL/kg, *p* = 0.475).

Finally, in the VCV study, lower PEEP during the VCV-PEEP_low_ phase was 7 [7–8] cmH_2_O, while VCV-PEEP_high_ was 12 [12–13] cmH_2_O (*p *< 0.001), both applied with protective tidal volume (7.0 [6.0–7.3] mL/kg vs. 6.8 [6.0–7.2] mL/kg; *p* = 0.94) and constant respiratory rate (18 [16–23] bpm).

### Validation of EIT-based MIF_glob_ vs. MIF_spiro_ and MEF_glob_ vs. MEF_spiro_

**Fig. 2:**
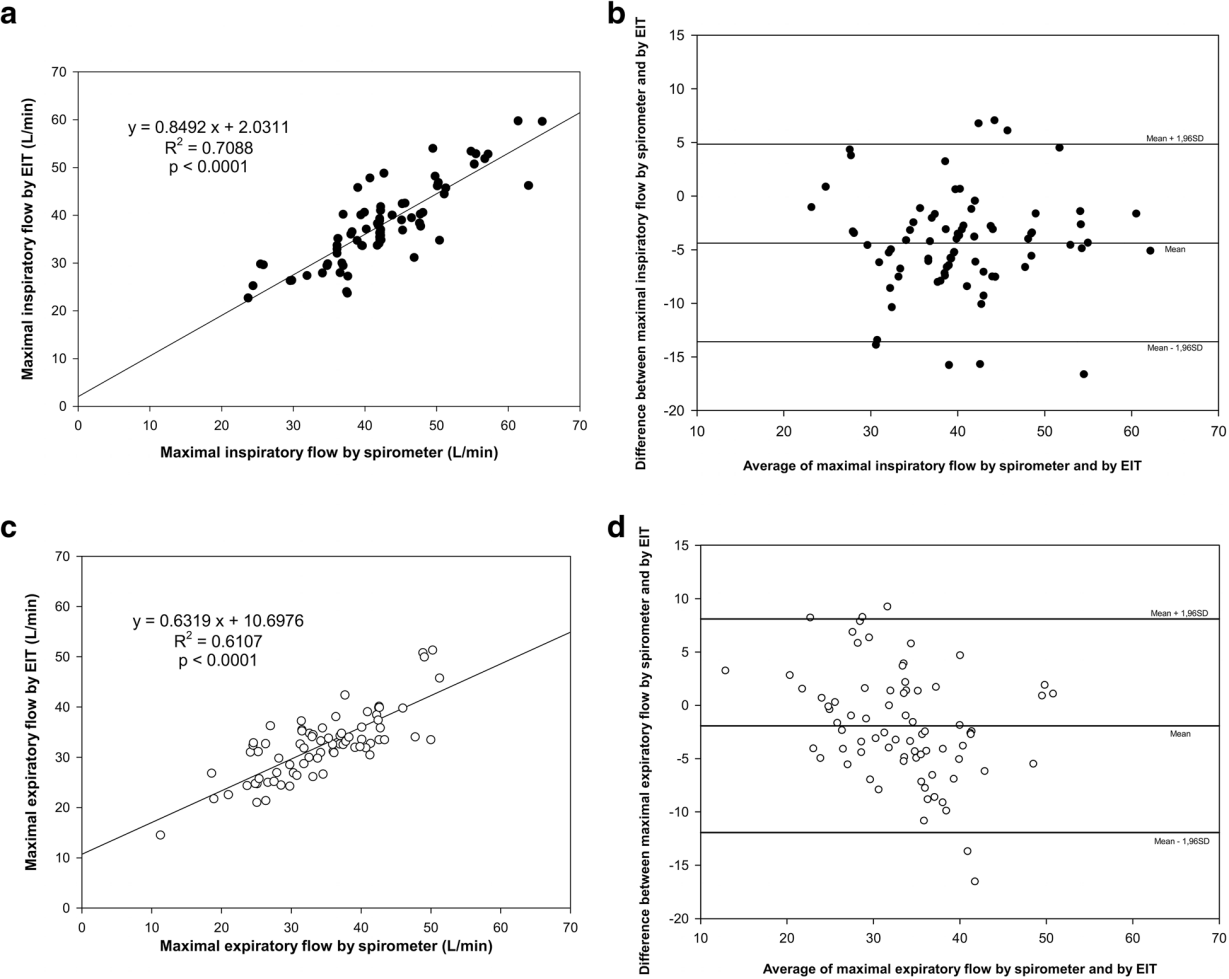
Correlations and Bland–Altman plots of airflows measured by spirometer and electrical impedance tomography (EIT). Top panels (**a**, **b**) show data for maximal inspiratory flow (MIF), while bottom panels (**c**, **d**) for maximal expiratory flow (MEF)

By pooling data from all six phases of both studies (*n* = 76 for each correlation), both MIF_glob_ and MEF_glob_ were tightly correlated with MIF_spiro_ and MEF_spiro_ (MIF_glob_ = 0.8 MIF_spiro_ + 2.0, *R*^2^ = 0.709 and *p* < 0.0001; MEF_glob_ = 0.6 MEF_spiro_ + 10.7, *R*^2^ = 0.611 and *p* < 0.0001) (Figs. 1 and 2) with clinically acceptable limits of agreement (mean bias for MIF = − 4.4 ± 4.7 L/min and limits of agreement − 13.6 to 4.8 L/min; mean bias for MEF = − 1.9 ± 5.1 L/min and limits of agreement − 11.9 to 8.1 L/min) (Fig. 2).

Regressions and Bland–Altman plots reporting specific results from each step of both studies (six conditions, 12 linear regressions and 12 Bland–Altman plots) can be found in the supplemental data (Additional file 1). Briefly, *R*^2^ values ranged between 0.606 and 0.776, all showing statistically significant correlations. Limits of agreement ranged between − 18.5 and 10.8 L/min, thus being clinically acceptable. Mean differences showed slightly lower values of MIF_glob_ and MEF_glob_ vs. spirometry data, except for differences between MEF_glob_ and MEF_spiro_ in the PSV_low_ and PSV-PEEP_low_ phases when a slight overestimation was observed.

### Effects of higher vs. lower pressure support and PEEP on regional homogeneity of maximal airflows

**Fig. 3:**
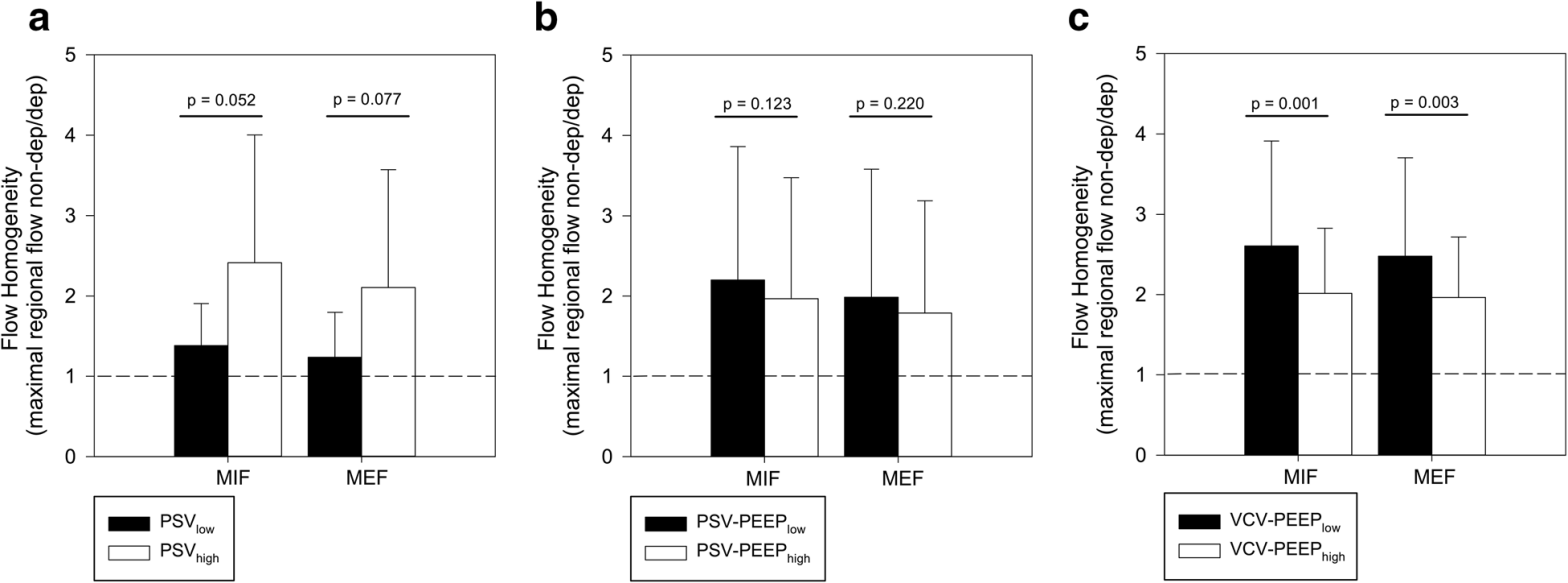
Lower levels of pressure support improved homogeneity of regional maximal flow distribution both during inspiration and expiration in comparison with higher support (PSV_low_ vs. PSV_high_) (**a**); higher positive end-expiratory pressure level led to an improvement in maximal inspiratory and expiratory flows (MIF and MEF) homogeneity both during pressure support ventilation (PSV-PEEP_high_ vs. PSV-PEEP_low_) (**b**) and volume-controlled ventilation (VCV-PEEP_high_ vs. VCV-PEEP_low_); (**c**) dashed line represents perfect homogeneity

The effect of lower pressure support on the homogeneity of regional distribution of maximal inspiratory and expiratory airflows was not statically significant (Fig. 3), in comparison with higher support (Table 2).

**Table 2:** Effects of higher vs. lower pressure support and PEEP on regional airflows and GI index measured by electrical impedance tomography (EIT)

	PSV_low_	PSV_high_	*p* value	PSV-PEEP_low_	PSV-PEEP_high_	*p* value	VCV-PEEP_low_	VCV-PEEP_high_	*p* value
MIF_non-dep_, L/min	19.5 ± 5.9	26.8 ± 9.7	*0.011*	26.7 ± 9.4	26.7 ± 10.9	0.969	26.2 ± 6.5	23.8 ± 5.6	*< 0.001*
MIF_dep_, L/min	15.3 ± 4.7	13.6 ± 5.5	0.286	15.9 ± 7.0	18.1 ± 8.6	0.339	11.4 ± 4.1	12.9 ± 3.9	*< 0.001*
MEF_non-dep_, L/min	15.5 ± 5.2	23.0 ± 10.1	*0.015*	22.7 ± 9.0	20.4 ± 9.6	*0.007*	21.6 ± 4.9	20.1 ± 4.6	*< 0.001*
MEF_dep_, L/min	14.1 ± 5.3	12.6 ± 3.6	0.422	15.4 ± 7.1	15.0 ± 6.2	0.819	10.0 ± 3.8	11.1 ± 3.6	*0.002*
GI index, %	57 ± 14	56 ± 9	0.798	58 ± 12	54 ± 10	*0.005*	52 ± 8	48 ± 8	*< 0.001*

PSV, pressure support ventilation; VCV, volume-controlled ventilation; MIF, maximal inspiratory flow; MEF, maximal expiratory flow; dep, dependent; non-dep, non-dependent; GI, global inhomogeneity

Application of higher PEEP improved maximal airflows homogeneity, albeit nonsignificantly, in the PSV study (Fig. 3), while the improvement was more consistent and highly significant in patients undergoing VCV. Higher PEEP during VCV increased airflows homogeneity by obtaining significantly higher MIF and MEF in the dependent lung regions and lowering them in the non-dependent ones.

GI index for regional tidal volume was significantly reduced only by higher levels of PEEP, both during PSV and VCV (Table 2). Interestingly, flow homogeneity was not correlated with the GI index (MIF homogeneity and GI, *R*^2^ = 0.018, *p* = 0.236; MEF homogeneity and GI: *R*^2^ = 0.017, *p* = 0.253).

By pooling data from all six phases of both studies (*n* = 80 for each correlation), regional MIF and MEF values were correlated with regional tidal volume (Vt) (Additional file 1: Figure S7), with *R*^2^ = 0.691 for MIF_non-dep_ vs. Vt_non-dep_; *R*^2^ = 0.585 for MIF_dep_ vs. Vt_dep_; *R*^2^ = 0.514 for MEF_non-dep_ vs. Vt_non-dep_ and *R*^2^ = 0.508 for MEF_dep_ vs. Vt_dep_ (*p* < 0.001 for all).

## Discussion

Study main findings can be summarized as follows: EIT may represent an accurate method to continuously monitor global maximal inspiratory and expiratory flows at the bedside, both during assisted spontaneous breathing and controlled ventilation; regional homogeneity of maximal airflows distribution might be enhanced by higher PEEP during VCV.

In the present study, we compared measures of maximal inspiratory and expiratory flows obtained by standard spirometry integrated in the ICU ventilator vs. a noninvasive method based on EIT monitoring in intubated hypoxemic patients undergoing both assisted and controlled ventilation. Spirometry represents the current clinical standard to monitor MIF and MEF [14] but it has limitations, especially when applied to patients undergoing noninvasive ventilation via face mask for the risk of air leaks. Moreover, spirometry cannot be performed in patients undergoing support by high-flow nasal cannula [15] because it would interrupt treatment by sealing the nares and it would interfere with normal breathing pattern by using a mouthpiece. We showed tight correlations between the two methods and clinically acceptable limits of agreement. Our findings in hypoxemic patients are in line with those reported by Bodenstein et al. [8] in healthy and lung lavage pigs ventilated in pressure-controlled ventilation mode. Like data coming from animals, we also showed that EIT modestly underestimates airflows values. This might relate to the different site where the spirometer and the EIT measure airflows: the first lies within the ventilator while EIT conceptually performs spirometry within the lungs. Thus, the airflows measured by the spirometer and EIT might effectively differ, with EIT potentially assessing the physiology of the distal airways, where broncho-constriction/dilation takes place. We showed tight correlations between MIF and MEF measured by EIT vs. spirometry also during pressure support ventilation: this was not assessed in the animal study by Bodenstein et al. [8], and given the recent interest in promoting protective spontaneous breathing in hypoxemic patients [16], our data might have increased the clinical relevance of airflows monitoring by EIT. Considered altogether, our and previous data suggest that EIT might represent a valuable addition to advanced respiratory monitoring of hypoxemic patients, yielding bedside noninvasive continuous assessment of airflows. This might be particularly relevant to understand patients’ severity, guide noninvasive support and verify the effects of therapy. Indeed, airflows measured by EIT have already been previously reported in non-intubated patients with chronic obstructive pulmonary disease (COPD) [17, 18], asthma [4] and cystic fibrosis [19] to stratify the degree of lung disease and to assess the response to diagnostic tests or to bronchodilator therapies. Finally, preliminary results showed that, in spontaneously breathing patients supported by Nasal High Flow, noninvasive regional MIF and MEF measured by EIT might be of relevance to detect reduced respiratory effort and improved mechanics, potentially decreasing the risk of patient self-inflicted lung injury (P-SILI) [20]. However, in the absence of appropriate calibration, during Nasal High Flow, EIT-based measure of airflows could only be assessed as changes from a given baseline.

In addition to accurate measure of global MIF and MEF, we showed that EIT can detect changes in regional airflows induced by different ventilation settings. This was not explored in the previous study by Bodenstein et al. [8] in animals with lung injury. Inhomogeneity in the spatial distribution of lung damage is a hallmark of AHRF and ARDS, due to gravitational distribution of lung edema, local intensity of inflammation and occlusion of segmental bronchi by secretions [21]. Moreover, interaction between mechanical ventilation and lung heterogeneity has been hypothesized as a major determinant of ventilation-induced lung injury (VILI), even in the presence of protective recommended settings. To this end, the role of airflows in inducing VILI has been largely neglected [22]. In the present study, we described that, during VCV, higher level of PEEP yielded more homogenous maximal airflows distribution between non-dependent and dependent lung regions. During PSV, higher PEEP ameliorated homogeneity but by less significant extent, maybe because of small sample size or less severe population. Anyhow, these results might represent a new specific physiological benefit for the lungs of hypoxemic patients, for a few different reasons: increased MIF and MEF in the dorsal lung could indicate lower resistance due to stenting of airway closure [23, 24]; reduced MIF and MEF in the non-dependent lung could imply lower risks of regional cyclic inspiratory–expiratory hyper-stretch [25, 26]; and more balanced velocity of dependent and non-dependent airflows could reduce the cyclic stress of mid-lung structures at the interface between the two regions [27]. The mechanisms through which lower support and higher PEEP improved regional homogeneity of airflows distribution likely differed (enhanced contraction of the dependent region of the diaphragm vs. higher inflating pressure at end expiration) but both finally led to increased regional transpulmonary pressure in the dependent compressed lung [28–30]. We previously showed that higher PEEP and lower levels of PSV lead to more homogenous tidal volume distribution in the lungs [9, 11]. Although regional tidal volume and maximal flows were somehow correlated in our patients, previous studies showed that flows could have specific pathophysiologic significance on the risk of VILI, even in the presence of constant tidal volume [25, 31]. Moreover, modified ventilation settings might not yield the same effects on regional flows and volumes. For example, if PEEP is increased but it remains below the airway opening threshold, maximal inspiratory flow could increase due to the higher pressure gradient while regional tidal volume might be only minimally affected as it mostly depends from regional compliance. Our data might suggest that targeting more homogenous distribution of regional airflows could represent another sensitive method to titrate personalized ventilation settings which deserves further scrutiny.

Our study has relevant limitations. First, we performed a new analysis of data coming from two prospective randomized crossover physiological studies aimed at exploring the effects of pressure support on tidal volume distribution and at validating EIT-based measure of end-expiratory lung volume change against the helium dilution technique. Hence, power analysis was not specifically performed to explore differences in regional airflows during different ventilation settings. Low number of patients might also explain lack of statistical significance in some of the study comparisons. Second, patients’ population (i.e. patients with AHRF and ARDS) was enrolled based on a reasonable clinical definition but this might have introduced heterogeneity in terms of lung disease severity and regional lung mechanics. Third, EIT does not image the whole lung, while spirometry measures airflows coming from all lung regions. However, as tidal volume measured by EIT is validated against imaging methods covering the whole lung [30], the same correlation could be inferred for airflows. Fourth, we explored relatively narrow and low ranges of pressure support and PEEP, but these might represent more closely current clinical practice. Fifth, classically, MIF and MEF are volitional maneuvers, highly influenced by the cooperation of the patient. In the present study, instead, we assessed MIF and MEF under standardized settings of PSV and VCV to increase clinical significance for intubated and sedated ICU patients. Further studies should be performed to verify whether the presented correlations between EIT and spirometry remain valid in awake, non-intubated patients.

## Conclusions

In intubated patients with AHRF and ARDS undergoing controlled ventilation and spontaneous breathing, maximal inspiratory and expiratory flows can be measured accurately by EIT. This is an exploratory study, and further studies need to be performed to validate airflows measured by EIT. Moreover, higher PEEP yields higher regional inspiratory and expiratory airflows in the dorsal lung probably through improvement in regional mechanics and eventually leads to more homogenous distribution of lung-distending and -deflating airflows in controlled models. EIT might represent a useful method for noninvasive assessment of global and regional airflows. The use of EIT as a dynamic tool to measure regional dynamic mechanical behavior and guide personalized treatments could be the object of future studies.

## Additional file

**Additional file 1.** Regressions and Bland–Altman plots reporting specific results from each step of both studies (six conditions, 12 linear regressions and 12 Bland–Altman plots).

## Data Availability

The data that support the findings of this study are available from the corresponding author upon reasonable request.

## Abbreviations

AHRF: acute hypoxemic respiratory failure
ARDS: acute respiratory distress syndrome
EIT: electrical impedance tomography
ICU: intensive care unit
MEF: maximal expiratory flow
PEEP: positive end-expiratory pressure
MIF: maximal inspiratory flow
PSV: pressure support ventilation
SD: standard deviation
IQR: interquartile range
VCV: volume-controlled ventilation
VILI: ventilation-induced lung injury
GI index: global inhomogeneity index
P-SILI: patients’ self-induced lung injury
