# Supplementary material for: Noninvasive assessment of airflows by electrical impedance tomography in intubated hypoxemic patients: an exploratory study
Source: Ann Intensive Care. 2019 Jul 22;9:83. doi: 10.1186/s13613-019-0560-5 (PMC6646434; doi:10.1186/s13613-019-0560-5)
Supplement: Supplementary file 1 — Additional file 1. Regressions and Bland–Altman plots reporting specific results from each step of both studies (six conditions, 12 linear regressions and 12 Bland–Altman plots). [file 13613_2019_560_MOESM1_ESM.docx]

**Additional file 1**

**Non-invasive assessment of airflows by electrical impedance tomography in intubated hypoxemic patients: an exploratory study**

Tommaso Mauri, MD^1^; Elena Spinelli, MD^1^, Francesca Dalla Corte, MD^2^; Eleonora Scotti, MD ^1^; Cecilia Turrini, MD ^2^; Marta Lazzeri, MD ^2^; Laura Alban, MD ^2^; Marco Albanese, MD ^2^; Donatella Tortolani, MD ^2^; Yu-Mei Wang, MD ^3^; Savino Spadaro, MD, PhD^2^; Jian-Xin Zhou, MD ^3^; Antonio Pesenti, MD ^1^; Giacomo Grasselli, MD ^1^.

^1^ Department of Anesthesia, Critical Care and Emergency, Fondazione IRCCS Ca’ Granda Ospedale Maggiore Policlinico, University of Milan, Milan, Italy

^2^ Department of morphology, surgery and experimental medicine, Azienda Ospedaliera-Universitaria Arcispedale Sant'Anna, University of Ferrara, Ferrara, Italy.

^3^ Department of Critical Care Medicine, Beijing Tiantan Hospital, Capital Medical University, Beijing, China.

**Additional Matherials and Methods**

**Study Population.** In the pressure support ventilation (PSV) study [1] ten consecutive intubated patients recovering from the acute respiratory distress syndrome (ARDS) [2], admitted to the general and neurosurgical intensive care unit (ICU) of the university affiliated San Gerardo Hospital, Monza, Italy were enrolled after they were switched from controlled mechanical ventilation to PSV, as per clinical decision. Exclusion criteria were: age younger than 18 years old, pregnancy, contraindication to electrical impedance tomography (EIT) use (e.g., presence of pacemaker or automatic implantable cardioverter defibrillator), impossibility to place the EIT belt in the right position (e.g., presence of surgical wounds dressing), altered diaphragm function (e.g., hemidiaphragm paralysis), and severe cardiovascular instability. Diaphragm dysfunction was assessed by ultrasounds [3] when clinically (e.g., low maximal inspiratory pressure) or radiologically (e.g., hemidiaphragm supra-elevation) suspected. The institutional ethical committee approved the study, and informed consent was obtained.

In the volume controlled ventilation (VCV) study [4], twenty intubated patients with acute hypoxemic respiratory failure (AHRF) or ARDS admitted to the same hospital ICU were enrolled. Inclusion criteria were: patients deeply sedated and paralyzed as per clinical decision; PaO_2_/FiO_2_ ≤ 300 mmHg with clinical positive end-expiratory pressure (PEEP) ≥ 5 cmH_2_O. Exclusion criteria were: age < 18 years, severe hemodynamic instability, evidence of cardiogenic pulmonary edema, history of chronic obstructive pulmonary disease, pregnancy, contraindications to the use of EIT (see above), inability to place the EIT belt (see above). For both studies, diagnosis of ARDS was based on the Berlin definition [2].

**Study Protocol.** In the PSV study three different 20 minutes-ventilator settings were tested in random order, while FiO_2_, PSV inspiratory ramp, and inspiratory and expiratory triggers were left unchanged:

- Clinically selected PSV (PSV_clin_) and PEEP (PEEP_clin_) levels;
- PSV_clin_ and PEEP_high_ (i.e., PEEP_clin_ + 5 cmH_2_O);
- A high PSV level (defined as a PSV level associated with p0.1 < 2 cmH_2_O) was compared versus a low PSV level (associated with p0.1 ≥ 2 cmH_2_O). To this end, if p0.1 during PSV_clin_ was < 2 cmH_2_O, then PSV_clin_ was regarded as PSV_high_, and a new PSV_low_ was selected during this phase, at least 4 cmH_2_O lower than PSV_clin_ and set to obtain p0.1 ≥ 2 cm H_2_O. At the opposite, if PSV_clin_ was associated with p0.1 ≥ 2 cmH_2_O, then PSV_clin_ was regarded as PSV_low_ and a new PSV_high_ was selected during this step, at least 4 cmH_2_O higher than PSV_clin_ to achieve p0.1 < 2 cmH_2_O. PEEP was maintained at PEEP_clin_ level in both cases.

Then, formal comparisons were performed between the following phases:

1. Low support at clinical PEEP (PSV_low_) vs. higher support at clinical PEEP (PSV_high_);
2. Clinical support at low PEEP (PSV-PEEP_low_) vs. clinical support at higher PEEP (PSV-PEEP_high_).

In the VCV study, volume control ventilation was set as follows: tidal volume (Vt) 6–8 mL/kg of predicted body weight (PBW), clinical PEEP, FiO_2_ to obtain a SpO_2_ level >90 %, and respiratory rate (RR) set to obtain arterial pH of 7.30–7.45.

After checking for stability, two 20 minutes randomized crossover steps (i.e., all patients undergoing both phases) were performed with unchanged Vt, FiO_2_, and RR were not modified:

- Clinical PEEP
- Clinical PEEP + 5 cmH_2_O

Then, formal comparisons were performed between the following phases:

1. Protective VCV at low PEEP (VCV-PEEP_low_) vs. protective VCV at clinical PEEP + 5 cmH_2_O (VCV-PEEP_high_).

**References**

1. Mauri T, Bellani G, Confalonieri A, et al. Topographic distribution of tidal ventilation in acute respiratory distress syndrome: effects of positive end-expiratory pressure and pressure support. *Crit Care Med* 2013; 41(7):1664–1673.
2. The ARDS Definition Task Force: Acute respiratory distress syndrome. The Berlin definition. *JAMA* 2012; 307(23):2526–2533.
3. Gerscovich EO, Cronan M, McGahan JP, et al. Ultrasonographic evaluation of diaphragmatic motion. *J Ultrasound Med*  2001; 20(6):597-604.
4. Mauri T, Eronia N, Turrini C, et al. Bedside assessment of the effects of positive end-expiratory pressure on lung inflation and recruitment by the helium dilution technique and electrical impedance tomography. *Intensive Care Med* 2016; 42(10):1576-1587.

**Additional Figures**

**Additional Figure 1. Comparison between spirometer and electrical impedance tomography flow data during the PSV_high_ phase.**

**Additional Figure 2. Comparison between spirometer and electrical impedance tomography (EIT) flow data during the PSV_low_ phase.**

**Additional Figure 3. Comparison between spirometer and electrical impedance tomography (EIT) flow data during the PSV-PEEP_high_ phase.**

**Additional Figure 4. Comparison between spirometer and electrical impedance tomography (EIT) flow data during the PSV-PEEP_low_ phase.**

**Additional Figure 5. Comparison between spirometer and electrical impedance tomography (EIT) flow data during the VCV-PEEP_high_ phase.**

**Additional Figure 6. Comparison between spirometer and electrical impedance tomography (EIT) flow data during the VCV-PEEP_low_ phase.**

**Additional Figure 7. Correlation between regional flow and tidal volume measured by electrical impedance tomography (EIT).**
